# On Abscesses at the Verge of the Anus

**Published:** 1878-10

**Authors:** 


On Abscesses at the Verge of the Anus.—Verneuil (*L'Abetlle Medicale*) recommends that in opening such abscesses, when they are close to the rectum, the operation for fistula should be performed at the same time.

